# Supplementary material for: Oral Supplementation of Ozonated Sunflower Oil Augments Plasma Antioxidant and Anti-Inflammatory Abilities with Enhancement of High-Density Lipoproteins Functionality in Rats
Source: Antioxidants (Basel). 2024 Apr 26;13(5):529. doi: 10.3390/antiox13050529 (PMC11117701; doi:10.3390/antiox13050529)
Supplement: Supplementary file 1 [file antioxidants-13-00529-s001.zip › Supplementary Material.docx]

**Supplementary Materials**

**Supplementary material S1:**

*N*-ε-carboxylmethyllysine (CAS-No 941689-36-7, Cat#14580-5g), dihydroethidium (DHE, 104821-25-2, Cat #37291), and acridine orange (AO, 65-61-2, Cat#A9231), oil red O (Cat#O0625), and 2-phenoxyethanol (Sigma P1126; St. Louis, MO, USA), paraoxon-ethyl (Cat. No. 36186) were procured from Sigma–Aldrich (St. Louis, MO, USA). All other chemicals and reagents else otherwise stated were of analytical grade and used as supplied.

**Supplementary material S2:**

**Supplementation of different amounts of SO and OSO in rats**

The selection of the used dose (3g/kg BW/day) for the SO and OSO was based on the preliminary screening experiments where the oral supplementation of different doses of SO and OSO (1 to 5 g/kg BW/day) on rats (n=5/group) was tested for one week. The findings revealed diarrhea and the decline in BW in both SO and OSO-supplemented groups at the doses of 4 and 5 g/kg BW/day. Contrary to this no diarrhea and BW reduction was observed for the oral supplementation of 1-3g/kg BW/day of SO and OSO. Therefore, the dose (3 g/kg BW/day) was selected for the present study.

**Supplementary Figure S1:**

**
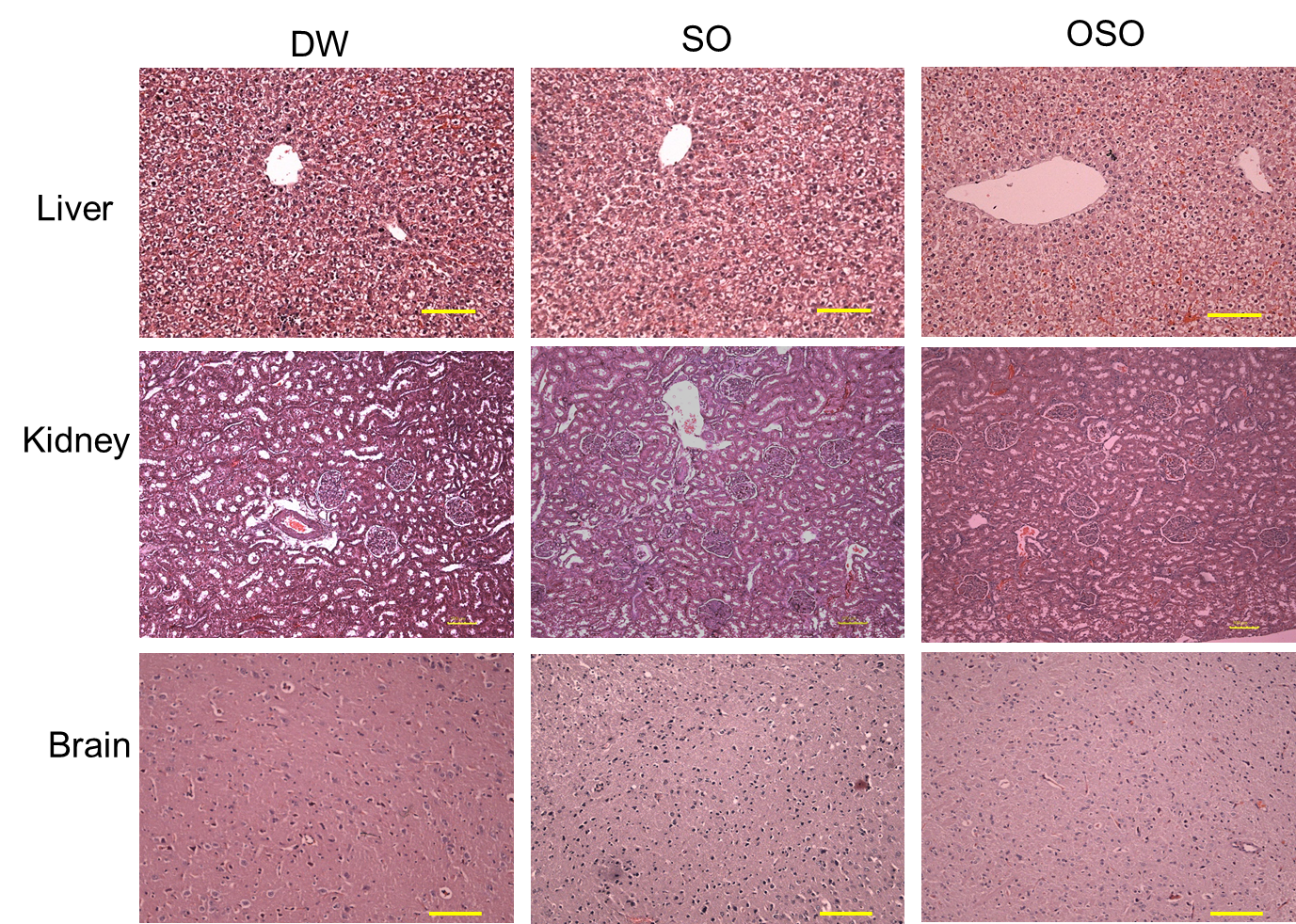
**

**Supplementary Figure S1:** Effect of the 4-week supplementation of sunflower oil (SO) and ozonated sunflower oil (OSO) on the hepatic, kidney, and brain histology examined by hematoxylin and Eosin (H&E) staining [Scale bar=100 μm].
